# Lifetime QALY prioritarianism in priority setting: quantification of the inherent trade-off

**DOI:** 10.1186/1478-7547-12-2

**Published:** 2014-01-14

**Authors:** Trygve Ottersen, Ottar Mæstad, Ole Frithjof Norheim

**Affiliations:** 1 Department of Global Public Health and Primary Care, University of Bergen, Kalfarveien 31, 5018 Bergen, Norway; 2 Chr. Michelsen Institute (CMI), Jekteviksbakken 31, Bergen, 5006, Norway

**Keywords:** Priority setting, Prioritarianism, Cost-effectiveness, Equity weights, Quality-adjusted life years, Political philosophy, Empirical ethics

## Abstract

**Background:**

Multiple principles are relevant in priority setting, two of which are often considered particularly important. According to the greater benefit principle, resources should be directed toward the intervention with the greater health benefit. This principle is intimately linked to the goal of health maximization and standard cost-effectiveness analysis (CEA). According to the worse off principle, resources should be directed toward the intervention benefiting those initially worse off. This principle is often linked to an idea of equity. Together, the two principles accord with prioritarianism; a view which can motivate non-standard CEA. Crucial for the actual application of prioritarianism is the trade-off between the two principles, and this trade-off has received scant attention when the worse off are specified in terms of lifetime health. This paper sheds light on that specific trade-off and on the public support for prioritarianism by providing fresh empirical evidence and by clarifying the close links between the findings and normative theory.

**Methods:**

A new, self-administered, computer-based questionnaire was used, to which 96 students in Norway responded. How respondents wanted to balance quality-adjusted life years (QALYs) gained against benefiting those with few lifetime QALYs was quantified for a range of different cases.

**Results:**

Respondents supported both principles and were willing to make trade-offs in a particular way. In the baseline case, the median response valued a QALY 3.3 and 2.5 times more when benefiting someone with lifetime QALYs of 10 and 25 rather than 70. Average responses harbored fundamental disagreements and varied modestly across distributional settings.

**Conclusion:**

In the specific context of lifetime health, the findings underscore the insufficiency of pure QALY maximization and explicate how people make trade-offs in a way that can help operationalize lifetime prioritarianism and non-standard CEA. Seen through the lens of normative theory, the findings highlight key challenges for prioritarianism applied to priority setting.

## Background

Available resources typically fall short of health care needs, making priority setting inevitable. Many priority-setting principles, prescribing how resources should be allocated, have been proposed. Of these, two general principles have been particularly prominent, although terminology has differed widely. According to the greater benefit principle, also known as the principle of health maximization, resources should be directed toward the intervention with the greater health benefit. According to the worse off principle, resources should be directed toward the intervention benefiting those initially worse off, i.e., those worse off without intervention. Either principle may have genuine weight—and independently influence practical reasoning—without necessarily outweighing all other considerations.

The greater benefit principle is blessed by an immediate appeal and enjoys support in both public opinion [1,2] and policy [3]. The principle is also intimately related to standard cost-effectiveness analysis (CEA) [4]. Despite criticism, the quality-adjusted life year (QALY) remains a popular specification of benefit and a popular cost-effectiveness unit for CEA [5,6]. The QALY is typically calculated by multiplying the number of years with a quality-adjustment factor, scaled so that 0 represents death and 1 represents “full” health. However, CEA with QALYs and the exclusive pursuit of health maximization have been heavily criticized, especially for making no direct reference to how benefits are distributed or to the plight of the worse off [7-12]. This feature is often considered fatal to the greater benefit principle as a standalone one.

The worse off principle speaks directly to this central deficiency of the greater benefit principle. Special priority to the worse off—often made explicit by reference to “need,” “severity,” or “urgency”—enjoys wide support in theory [7,9,13,14], public opinion [11,15], and health policy [3,16]. However, there is an ongoing debate about how “severity” and “the worse off” are best understood [9,16-18]. According to one reasonable specification, the worse off are those with the fewer lifetime QALYs, i.e., those who will have the fewer QALYs over their entire lifespan [7,16]. This comprehensive specification incorporates both quality and quantity of health as well as past, present, and future health. Assuming no uncertainty, lifetime QALYs can also be labeled quality-adjusted life expectancy (QALE).

The worse off principle is also insufficient by itself, as it makes no direct reference to benefits from intervention. A compromise between the greater benefit principle and the worse off principle is thus appealing because each principle responds to the primary deficit of the other. This compromise accords with so-called prioritarianism, whose central claim is that “[b]enefiting people matters more the worse off these people are” [13]. Derek Parfit’s description of how prioritarianism “contains the idea that benefits are good” and “merely adds that benefits matter more the worse off the people are who receive them” indicates how prioritarianism can be seen as the compromise between the greater benefit and the worse off principles [13]. While the greater benefit principle can motivate standard cost-effectiveness analysis (CEA) with QALYs, prioritarianism can motivate CEA with equity or distributional weights [7,10,19,20]. These weights specify the importance of health maximization relative to some other concern, for example, that for the worse off. This other concern can be integrated into CEA by multiplying the weights in question with the original cost-effectiveness unit.

A crucial question for the operationalization of prioritarianism is how its two inherent principles should be balanced. This may be seen as a particular equity-efficiency trade-off and to be at the center of multi-criteria decision analysis [21]. The trade-off between greater benefits and benefiting the worse off is often considered an object of reasonable disagreement [14]. With no consensus on a principled solution, the appropriate trade-off may be informed by priority judgments of the general public [14,22]. The empirical study of such judgments—as with empirical ethics more generally—can provide input to individual moral reasoning, public deliberation, and policy making [14,22,23].

Only a few studies [24-26] have quantified how people trade off QALYs gained against benefiting those with the lower QALE per se, and these studies were limited in important respects. Two of them [24,25] covered only the upper part of the QALE scale, and the third study [26] addressed the lower part only indirectly through rank or relative position of the interventions’ target groups. This is unfortunate because the lower part of the scale appears particularly controversial and relates to a contentious aspect of lifetime QALY prioritarianism [16,27]. In addition, two of the three studies [24,25] did not explore how average findings can harbor fundamentally opposed moral views, and one studies did so only in little detail [26]. Such deeply opposed views differ not only in how much more or less priority they attribute to different target groups but also in their ranking pattern, i.e., the very order of priority among those groups. Fundamental disagreement of this kind is not only interesting in its own right; it can also inform the proper use of aggregate data. Moreover, two of the studies [25,26] did not provide respondents with the distributional context of the interventions in question, i.e., the overall distribution of health in society, while the third study [24] presented respondents with choices only between entire societies, each consisting of only two different groups. This is suboptimal given that normative theory tends to stress the relevance of several aspects of the background distribution [28-30].

The objective of the present study was to 1) develop and test a new, comprehensive questionnaire; 2) assess people’s support for lifetime QALY prioritarianism and quantify how they trade off the greater benefit principle and the worse off principle; and 3) examine the links between the empirical findings and normative theory. With respect to the trade-off, we specifically wanted to study trade-offs between QALYs gained and initial QALE across a wide range of QALE levels, assess underlying ranking patterns, and explore the relevance of distributional context.

## Methods

A comprehensive, self-administered, computer-based questionnaire was developed and used to elicit priority judgments from a convenience sample. The questionnaire was distributed by email to an entire class of approximately two hundred first-year medical and dental students in Bergen, Norway, in 2008. No monetary reward was offered, and ninety-six students responded. All respondents were attending a course on medical ethics and had generally no formal training in health economics or priority setting. Instructions were provided both in the questionnaire and in a preceding lecture. In both settings, emphasis was put on explaining key technical concepts, including QALYs. An additional Excel file includes a translated version of the entire questionnaire (see Additional file 1).

### Description and elicitation

Trade-offs were elicited using a common method, sometimes called “the gain trade-off technique” or “the benefit trade-off technique”. Several aspects of this method have been discussed theoretically [31,32], and variations of it were employed in the three trade-off studies discussed above [24-26]. The basic idea is that the respondent addresses pairs of interventions that vary in two dimensions: total health benefits and the level of a particular attribute of the target group. Through a series of binary choices or by direct question, the respondents indicate what interventions they consider equally important. By comparing the health benefits of these two interventions, we can infer how much benefit the respondents are willing to sacrifice to advantage someone with one rather than another level of the attribute in question.

**Figure 1:**
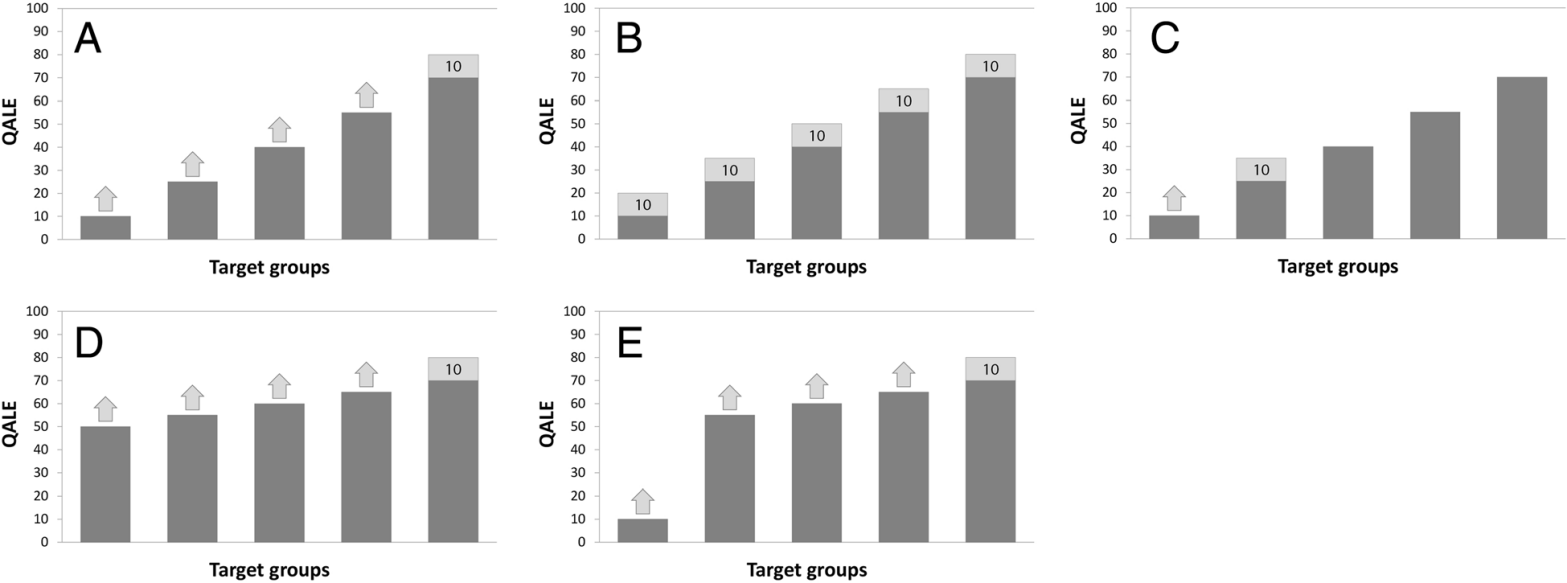
**A, B, C, D, and E panels representing tasks in the questionnaire.** QALE without intervention and QALYs gained from intervention are indicated by dark and light grey bars, respectively.

The part of the questionnaire reported here consisted of one main task (task A) and four supplementary tasks (tasks B to E). All tasks are illustrated in Figure 1 below. In the main task, interventions were characterized by the initial QALE of the target groups. The distribution of QALE in the hypothetical society was represented by five groups, each consisting of one fifth of the total population. Their absolute levels of QALE were 10, 25, 40, 55, and 70, respectively. These levels were chosen so as to cover a wide range of QALE levels and make equal the absolute difference between adjacent groups. Each intervention targeted one of the five groups. The reference intervention would provide, if implemented, a benefit of 10 QALYs to every member of the group with an initial QALE of 70. Respondents addressed four decision problems, each in which they compared that reference intervention with an alternative intervention. They were asked to state the number of QALYs gained from the alternative intervention that would make the two interventions equally important in terms of priority. In other words, respondents indicated their indifference value for each of the four pair-wise comparisons. Values permitted were 0.25, 0.5, 0.75, and every integer between 1 and 99. Respondents were specifically asked to adopt the perspective of a health planner concerned with doing what is best for society. Moreover, they were encouraged to assume that things other than initial QALE and QALY benefits were equal. More specifically, in the preceding lecture, respondents were told that the benefits and the beneficiaries were equal in all possible respects and examples were provided. In the questionnaire, this was emphasized by making explicit that the groups were of equal size, that the health benefits were similar except for the target group, and that the costs were the same. There were also repeated reminders that the health benefits were otherwise equal.

Task B was similar to the main task in most respects. The difference was that all the five interventions would provide, if implemented, a benefit of 10 QALYs to every member of their respective target group. Hence, there was no benefit trade-off involved. Respondents were asked to directly rank the interventions in order of what priority they should have for implementation. Task B was included to explore such direct rankings, as opposed to rankings only implied by responses to the main task. Tasks of the former kind are typically considered easier for the respondents and may therefore produce more valid and reliable information about ranking patterns than trade-off exercises [33,34]. Task B was included also in order to compare direct with implied rankings and thereby test intrarespondent consistency. Task B appeared at the end of the questionnaire, but an identical task was placed at the beginning. This latter task was included as a warm-up exercise and to test the extent to which responses changed during the exercise.

Task C was included in order to directly explore priority judgments with respect to the lower part of the QALE scale. The difference from the main task was that respondents directly compared the two groups with the lowest initial QALE, i.e., those with an initial QALE of 10 and 25. This was done because their relative importance indicated by such a task may differ from that inferred from comparisons between each of those two groups and the group with an initial QALE of 70. The reference intervention in task C targeted the group with an initial QALE of 25 and would provide, if implemented, a benefit of 10 QALYs to every member of that group. Respondents reported indifference values like in the main task.

Tasks D and E were included to explore the relevance of distributional context, i.e., the overall distribution of health in society. Accordingly, the initial levels of QALE across groups differed from the main task. In task D, initial QALE levels were 50, 55, 60, 65, and 70, respectively. Compared to the main task, this one involved a distribution with a higher average level of QALE and with smaller absolute differences between groups. This distribution is more representative for certain egalitarian societies, e.g., the Scandinavian countries, than the distribution in the main task. In task E, initial QALE levels were 10, 55, 60, 65, and 70, respectively. Compared to the main task, the worse off group here stands out more clearly. For example, the absolute difference between that group and the rest is much larger. Such a distribution can represent populations in which the ratio between child and adult mortality rates is particularly high, something that is characteristic of several low-income countries. Respondents reported their indifference values like in the main task.

### Calculation of distributive weights

Distributive weights were calculated for the main task and tasks C to E. For each pair-wise comparison, we took the indifference value *x* to imply that the respondent was indifferent between providing 10 QALYs to someone at the reference level of initial QALE and providing *x* QALYs to someone at the alternative level. The distributional weight for the alternative level can then be defined as the ratio between the benefit in the reference case and the indifference value *x*, i.e., 10/*x*[32,35]. In other words, the distributional weight indicates how many times more important a QALY is when benefiting someone at that level. While we here will follow most of the literature in taking these weights as adequate approximations of marginal weights, i.e., weights to be applied to the benefit of 1 QALY, marginal and nonmarginal weights can certainly differ [32]. Mean and median weights for every pair-wise comparison were calculated from all responses that met the inclusion criteria described below.

### Analysis of ranking patterns

In contrast to sets of distributional weights, ranking patterns only provide information about the *order* of priority. Responses from tasks A and B were classified into ten ranking patterns. These patterns describe how ranking changes with initial QALE when seen from below and with increasing rank, i.e., importance, along the y-axis. They were labeled as follows: 1) monotonically decreasing; 2) monotonically increasing; 3) constant; 4) inverted-U shape with peak at the second lowest group; 5) inverted-U shape with peak at the middle group; 6) inverted-U shape with peak at the second highest group; 7) U-shape with base at the second lowest group; 8) U-shape with base at the middle group; 9) U-shape with base at the second highest group; and 10) other. Certain adaptations were made, partly motivated by the fact that response options between 1 and 99 in task A were limited to integers. If a respondent gave two adjacent groups an equal rank, the group with the highest ranked neighbor was classified as the one with the higher rank. If the two groups were located at one end of the QALE scale, the group at the very end was classified as the one with the higher (lower) rank if the middle group had a lower (higher) rank than the two equally ranked groups.

As indicated, individual rankings were also used to assess intrarespondent consistency. Responses to tasks A and C to E were outright excluded for respondents who ranked one group highest in task A and lowest in task B or vice versa. Exception was made if that group shared rank with three or more other groups. Tasks A and C to E were also excluded for respondents who had not completed both task A and B, as consistency could not be assessed. This was the case for 20 respondents. A given task was considered complete only if all four interventions had been ranked, directly or indirectly. In total, the initial inclusion criteria for tasks A and C to E were met for 66 respondents. The final inclusion of each particular task also required that that specific task was complete. For task B—which involved no trade-offs—no initial inclusion criteria had to be met, and it was sufficient that that very task was complete. This was the case for 93 respondents.

## Results

**Figure 2:**
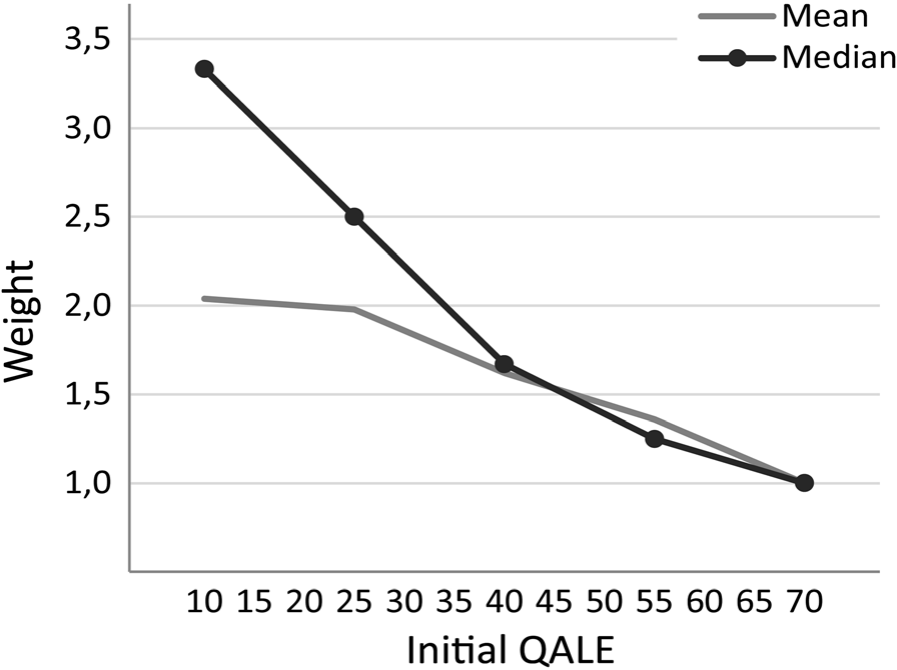
Mean and median distributional weights from the main task (A).

Figure 2 shows mean and median distributional weights for different levels of initial QALE. Median (mean) weights were 3.3 (2.0), 2.5 (2.0), 1.7 (1.6), 1.3 (1.4), and 1.0 (1.0) for initial QALE levels of 10, 25, 40, 55, and 70, respectively. This indicates, among other things, that the median response valued a QALY 3.3 or 2.5 times more when benefiting someone with an initial QALE of 10 or 25 rather than 70. The median, as well as mean, distributional weights decrease monotonically with initial QALE. In other words, for any two groups, a lower initial QALE implies higher weight. To that extent, the sets of mean and median responses correspond with prioritarianism.

### Rankings

**Figure 3:**
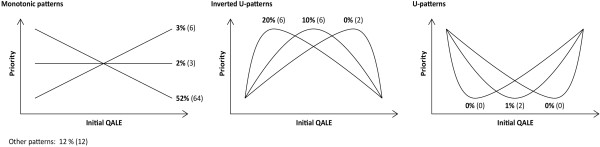
Relative frequencies of different ranking patterns in task B (and in task A in parentheses).

Figure 3 shows the relative frequencies of different ranking patterns in task B as well as in the main task (A). While such patterns in task B follow from direct rankings by the respondents, patterns in task A follow from rankings implied by the respondents’ indifference values. Most frequent was a monotonically decreasing pattern, reported by 52% of the respondents in task B and 64% in task A. Second most frequent was an inverted-U pattern with peak at the second lowest group, reported by 20% and 6%, respectively. In both tasks, 12% of the responses did not fit any of the nine specific patterns and were classified as “other”. As for Figure 3, illustrations of nonmonotonic patterns need not represent the exact internal relation between two groups located at opposite sides of a peak or a base.

As described, 52% and 64% of the respondents reported a monotonically decreasing pattern. This indicates not only that the sets of mean and median distributional weights accord with prioritarianism, but also that the majority of individual ranking patterns do. Nonetheless, the many competing ranking patterns that were reported expose a fundamental conflict underlying the observed mean and median weights. Not only was there division about how much more weight the favored groups should have; there was also a much deeper disagreement about what groups should be favored at all. As suggested by the relative frequencies in Figure 3 and further discussed below, the lower end of the QALE scale is the primary source of this disagreement.

**Figure 4:**
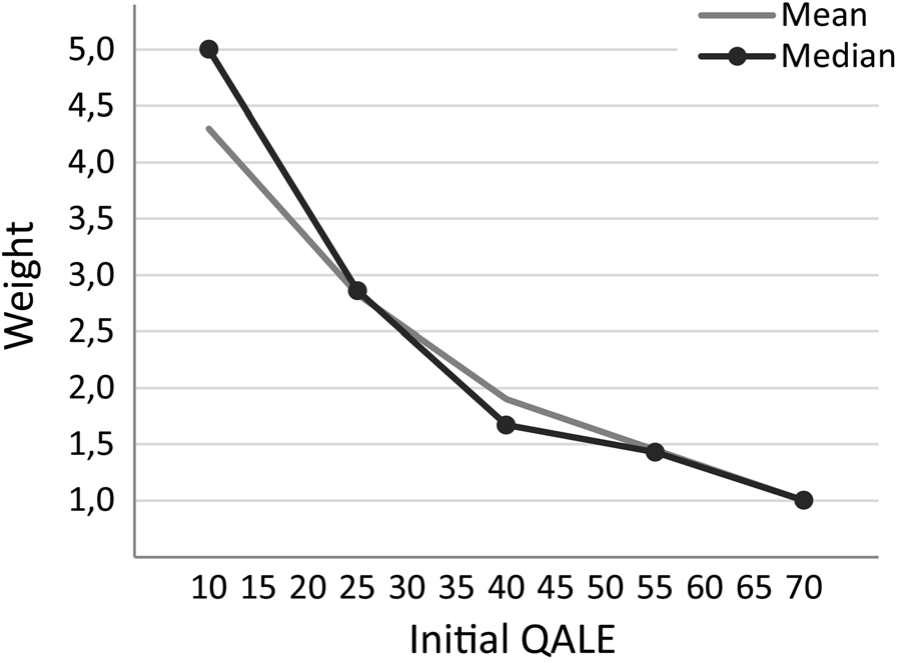
Mean and median distributional weights from the main task (A) among respondents with weights monotonically decreasing with initial QALE.

Given that the weights in Figure 2 are aggregates over very different ranking patterns, it is interesting to also consider mean and median weights among those who actually reported a monotonically decreasing ranking pattern in the main task. These sets of weights are shown in Figure 4. Median (mean) weights were 5.0 (4.3), 2.9 (2.8), 1.7 (1.9), 1.4 (1.5), and 1.0 (1.0) for initial QALE levels of 10, 25, 40, 55, and 70, respectively. Compared to Figure 2, the corresponding weighting function is, not surprisingly, much steeper.

In addition to task B, which appeared at the end of questionnaire, respondents addressed an identical task in the beginning. The change across the two tasks was modest for the sample as a whole. The maximum absolute change in relative frequency for any pattern was 2%. At the individual level, however, 20% of the respondents revised their ranking pattern.

### Priority with respect to low levels

Priority judgments with respect to the lower and supposedly most controversial part of the QALE scale were examined in several ways. For the main task, the implied mean and median distributional weights for an initial QALE of 10, with 25 as the comparator, were 1.0 and 1.3, respectively. For the direct comparison in task C, mean and median weights were both 1.3. Also with respect to this lower part of the QALE scale, we explored fundamental disagreements in terms of ordinal rank. The proportion of respondents who gave some priority to an initial QALE of 10 over 25 was 61% in task A, 47% in task B, and 68% in task C. Equal priority was reported by 17%, 14%, and 11% of the respondents, respectively. Overall, these findings suggest that a majority of people are prioritarians also with respect to the lower end of the QALE scale, but the findings also suggest that the disagreement on this issue is indeed substantial.

### Impact of distributional context

**Figure 5:**
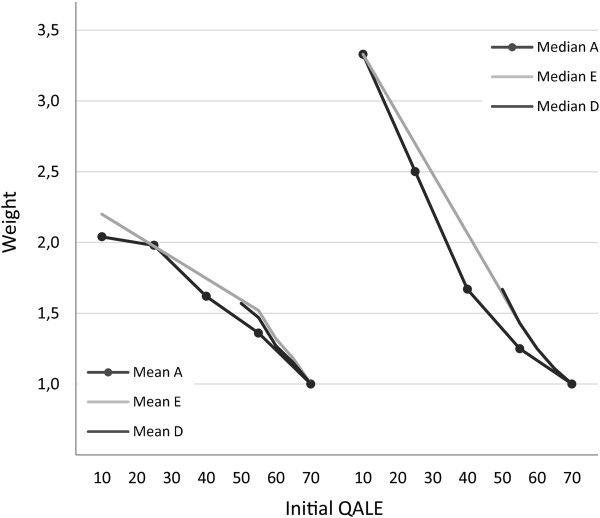
Mean and median distributional weights from the main task (A) and tasks D and E.

Figure 5 shows how distributional weights for specific target groups varied with distributional context, i.e., the overall distribution of health in society. The relevant distributions are illustrated in Figure 1. While the variation was modest, some differences can be seen. For example, the group with an initial QALE of 10 was assigned the same median weight in tasks A and E, but the mean weight was slightly higher (0.16 units) in task E. One possible explanation is that the worst off group stood out more clearly in task E. For example, in that task there was a much larger absolute and relative difference between the worst off group and the adjacent group as well as a larger difference between the worst off group and the population average [28-30]. As for the group with an initial QALE of 55, mean and median weights were higher in task D (0.11 and 0.18 units) and task E (0.16 and 0.18 units) than in task A. One possible explanation is the variation in rank or relative position of that group across tasks [28-30]. In task A, the group with an initial QALE of 55 was next to the best off, while in tasks D and E, that group was next to the worst off. Overall, these findings suggest that the weights found are quite robust across the distributional contexts explored. To the extent that there was some variation, this indicates that different reasons for priority to the worse off may be operating. This will be further discussed below.

## Discussion

The findings of this study shed light on lifetime QALY prioritarianism with respect to both justification and specification.

The findings suggest that *both* the greater benefit principle and the worse off principle are important, thereby providing basic support for prioritarianism. An exclusive concern for health benefit maximization would imply an indifference value of 10 QALYs—corresponding to a distributive weight of 1—for every group. Only two respondents reported such values in the main task. All others found initial QALE relevant and were willing to sacrifice some health benefits. More specifically, the findings suggest that this concern for initial QALE mainly has a prioritarian pattern, i.e., the value of a QALY decreases monotonically with initial QALE. This pattern was exhibited both by the sets of mean and median distributional weights and by the majority of individual ranking patterns. At the same time, the findings do not indicate an exclusive concern for the worse off as only one respondent reported the lowest possible indifference value (0.25 QALYs) for any group. Overall, then, people seem to support both the greater benefit and the worse off principles and appear willing to trade off the two principles. This central trade-off was further specified by distributive weights, which express quantitatively the willingness to sacrifice total benefits for the sake of benefiting the worse off. Weights of this kind can help operationalize prioritarianism and provide input to CEA with weighted QALYs.

The main limitation of the study relates to the size and composition of the sample used. The sample was relatively small and involved only medical and dental students. The findings thus need not be representative for the general public in Norway or elsewhere. It is also worth noting that lifetime QALY prioritarianism can be defended on several fronts and that not all were addressed in the present study. Our findings provided empirical support to lifetime QALY prioritarianism primarily in two ways: by favoring a principle sensitive to lifetime QALYs over pure QALY maximization and by favoring a monotonically decreasing priority pattern across initial lifetime QALYs over other patterns. The findings do not, however, defend lifetime QALY prioritarianism against every alternative specification of prioritarianism. In particular, people may favor a time-specific view over a lifetime view [16,17].

Another aspect of the responses to initial QALE is that we cannot know exactly what factors respondents considered to be associated with QALE. While they were told that interventions were equal in every possible respect apart from QALY gain and initial QALE, respondents may have assumed, for example, that a low QALE is associated with being disadvantaged also in other respects. As for benefits, it is important to note that the tasks involved fairly large health gains, i.e., at minimum 10 QALYs for at least one group. This was done to ensure that the respondents mainly dealt with benefits they considered significant. At the same time, inferences from choices over large benefits to claims about the relative importance of marginal health gains must be made with care. One reason is that nonmarginal benefits span several levels of health, and the distributional concerns involved may deviate from those related only to a marginal benefit [32]. In addition, the value people assign to benefits appears to be nonlinear in QALYs—possibly involving thresholds—in a way that goes beyond concerns for initial level of health [36,37]. Accordingly, the distributive weights could have been different if the reference value was of a different size.

For all studies eliciting stated judgments or preferences, it is also well known that responses can be highly sensitive to minute details in presentation and framing [38,39]. When designing the questionnaire, we therefore paid much attention to how tasks and questions were described. This effort included pilot sessions in which respondents explained how they interpreted the questionnaire. Nevertheless, of those 86 respondents who completed both task A and B, 20 (23%) did not meet the inclusion criteria. Part of the explanation is probably that in task A, *lower* indifference value implied *higher* importance. It was indeed this feature that some pilot participants found counterintuitive and that seemed to partly explain why some of their direct rankings and implied rankings conflicted. Both the preceding lecture and the final questionnaire therefore put considerable effort in making respondents aware of that property. The fact that a highly educated sample still found the trade-offs so challenging suggests that certain adaptations would be needed if the questionnaire was to be used with a sample of the general public. Alternative modifications include a different way of expressing trade-offs, use of less abstract interventions, use of face-to-face interview modes, and some combination. The changes in responses across task B and the identical task also highlight that people are unlikely to have ready-made answers and that respondents should be given considerable time for individual or group deliberation [40,41].

Despite the limitations, the findings of this study can be relevant in multiple ways. As with empirical ethics more generally, the findings can help identify key moral issues or make such issues more concrete. More specifically, beyond the general findings discussed above, this study highlighted at least three particularly challenging issues for prioritarianism in priority setting as well as for priority setting more generally. One issue was accentuated by the interindividual variation in ranking patterns and the fundamental moral disagreements underlying mean and median weights. In such a situation, does it make sense to use these weights for priority setting? This question calls attention both to the aggregation of individual judgments within priority setting and to the complex relation between empirical ethics and normative theory [14,22,23,42]. As for the latter, how can public opposition affect the theoretical case for a prioritarian pattern, the appropriateness of that case guiding policy, or both? Conversely, how is moral theory to constrain empirical ethics? It can be argued, for example, that public judgments should affect priority setting only within some narrow confines set by principled arguments. If so, one could ask whether the prioritarian pattern is theoretically justified to the extent that the role of empirical ethics is only to inform its slope.

This study also emphasized a particularly contentious aspect of lifetime QALY prioritarianism: monotonically decreasing priority at the very lower part of the QALE scale. We compared groups with initial levels of QALE of 10 and 25 in several ways and found considerable disagreement. As described above, respondents were split almost evenly on whether the former group should have priority. Age is a major determinant of QALE, and the opposition to prioritizing those with very low QALE plausibly derives from an unwillingness to prioritize the very young. For example, a challenging case for lifetime QALY prioritarianism with respect to age is the following. Two terminally ill patients will gain 5 QALYs from intervention and are similar in all possible respects but their age. One patient is an infant; the other is 25 years old. If we can treat only one, lifetime QALY prioritarianism favors the infant, something which runs counter to intuitions commonly held according to some empirical studies [11,43]. Priority to adolescents and young adults over infants and children also follows from certain theoretical frameworks for priority setting [44] and from the age weighting that previously underlay disability-adjusted life years (DALYs) [45]. Moreover, several rationales for such priority have been offered [19,27,43,45-47]. In light of this skepticism toward priority to the very young, both in theory and public opinion, priority with respect to very low levels of QALE is a pressing challenge for priority setting and for lifetime QALY prioritarianism in particular. The fact that low QALE does not to need imply low expected age of death, if quality is sufficiently low, makes the issue even more complex and relevant for future empirical and theoretical inquiry. In particular, it would be interesting to understand how respondents reason when confronted with initial health in terms of a certain level of QALE. What do they assume is the relative contribution of quality and quantity to that construct, and how do they subsequently apply their view about the relative importance of the two?

Another important issue was highlighted by the modest, yet palpable variation in weights across distributional contexts: the issue of reasons for priority to the worse off. Some, including Parfit, deem necessary for prioritarianism that the worse off have priority because they are at a lower absolute level. However, many other, partly overlapping reasons exist [9,13,26,28,30,48]. A key distinction is that between reasons that are directly relational and those that are not [9,13]. Central among the latter is the reason just mentioned: that the worse off are at lower *absolute* level [13]. In contrast, relational reasons—of which egalitarian reasons are typically considered a subset—are concerned with how people fare *relative* to others. Potentially relevant relations include rank or relative position, absolute difference, and relative difference or ratio [28-30].

Despite Parfit’s emphasis on absolute level, some broader conceptions of prioritarianism allow for a variety of reasons to motivate priority to the worse off. Such a conception is particularly useful when prioritarianism is contrasted with the sole operation of the greater benefit principle or pure health maximization. Given a broad conception of prioritarianism, variation in distributive weights across distributional contexts also becomes more interesting. While the variation in this study was modest, the variation found allows for some hypotheses based on theoretical work [28-30]. The variation across tasks A and E, for example, suggests that even for a given absolute level of QALE, priority to the worse off group will vary with the group’s absolute and relative difference from other groups in society. Moreover, the difference between task A versus tasks D and E indicates that also relative position or rank can change priorities independent of absolute QALE level. Overall, the modest variation suggests that distributive weights can be quite robust across distributional contexts and that absolute level of QALE may be the dominant reason for priority to the worse off. Both this hypothesis and the variation across contexts need to be further examined, and this calls for more research into what reasons drive people’s concern for the worse off in health.

## Conclusions

The present study used a new, comprehensive questionnaire to assess people’s support for lifetime QALY prioritarianism and to quantify how they trade off QALYs gained against benefiting those with low initial QALE. In addition, the study carefully examined the links between the findings and normative theory.

Support for lifetime prioritarianism was found in mean and median weights as well as in individual ranking patterns, and we calculated distributional weights of a kind that can make prioritarianism more operational and provide input to non-standard CEA. When applying the lens of normative theory, several challenges to lifetime QALY prioritarianism became apparent. These concern the aggregation of weights across conflicting ranking patterns, priorities with respect to low QALE levels, and the relevance of distributional context. These challenges are important topics for future empirical as well as theoretical research.

The limited and nonrepresentative sample of respondents restricts direct policy recommendations. However, the findings of this study add to the evidence that pure QALY maximization and standard CEA with QALYs are insufficient bases for priority setting—this time in the context of lifetime health. The findings should encourage researchers as well as policy makers to pay more attention to lifetime QALY prioritarianism. The study also indicates how people specifically want to balance the concerns for QALYs gained and initial QALE, but larger and more representative studies are certainly needed. When the appropriate balance is better established, the difference between pure QALY maximization and lifetime QALY prioritarianism will also stand out more clearly.

## Consent

Every respondent was informed prior to the study and gave written, informed consent to participate.

## Abbreviations

CEA: Cost-effectiveness analysis; DALY: Disability-adjusted life year; QALE: Quality-adjusted life expectancy; QALY: Quality-adjusted life year.

## Competing interests

We declare that the authors have no competing interests.

## Authors’ contributions

All authors participated in the design of the study and in the interpretation of results. TO collected data and drafted the paper. All authors read and approved the final manuscript.

## Supplementary Material

Additional file 1 Questionnaire translated into English. Click here for file
